# Passive removal of silicone oil through 23 gauge transconjunctival sutureless vitrectomy system

**DOI:** 10.12669/pjms.323.9498

**Published:** 2016

**Authors:** Nisar Ahmed Siyal, Lakhani Das Hargun, Shahid Wahab

**Affiliations:** 1 Nisar Ahmed Siyal, Assistant Professor, Ophthalmology Unit I, Dow University of Health Sciences & Civil Hospital, Karachi, Pakistan; 2 Lakhani Das Hargun, Assistant Professor, Ophthalmology Unit I, Dow University of Health Sciences & Civil Hospital, Karachi, Pakistan; 3 Prof. Shahid Wahab, Professor, Ophthalmology Unit I, Dow University of Health Sciences & Civil Hospital, Karachi, Pakistan

**Keywords:** Passive removal of silicone oil, Suture less vitrectomy

## Abstract

**Objective:**

To study the outcomes of passive removal of silicone oil by 23 Gauge Transconjunctival Sutureless Vitrectomy System.

**Methods:**

This prospective, consecutive case series study was conducted at Ophthalmology Department Unit I, Dow University of Health Sciences, Civil Hospital Karachi from January 2011 to December 2014. Only psuedophakic eyes with silicone oil temponade were selected. Main outcome measures were intra ocular pressure, time taken for removal of silicone oil, per operative and post operative complications. Pre and post operative IOP was compared by using two-tailed paired t-test and mean values with standard deviation were computed using difference of 95% confidence interval. Chi square test was applied for correlation of different variables. P-value of less than 0.05 was considered statistically significant.

**Results:**

Out of 79 patients who underwent passive ROSO, 38 (48.1%) were males. Mean age of patients was 47.5±7.1 (sd) years. Mean time taken for passive ROSO was 7.31±2.41 (sd) minutes. Pre and post operative intra ocular pressure shows statistically significant (p=0.000) decrease in IOP. Retinal redetachment found in 13 (16.5%) cases during follow up period.

**Conclusion:**

Passive removal of silicone oil with 23 G suture less vitrectomy system is safe and effective in terms of less per operative and post operative complications. In this simple technique, there is less tissue trauma and little time consumed so it provides more comfort to patients and surgeons as well.

## INTRODUCTION

Silicone oil is a liquid with unique properties of surface tension. It had been widely used as long term internal temponading agent during retinal re attachment surgery by vitreo retinal (VR) surgeons for the last almost six decades.^1^ However, silicone oil in long term not only can be toxic to photoreceptors^2^ but also cause other complications such as cataract, secondary glaucoma and keratopathy as well.^3^,^4^ Therefore removal of silicone oil (ROSO) becomes essential in several cases and even some surgeons recommend it in almost all cases with few exceptions. Few VR Surgeons advocate ROSO as early as a month or few weeks after initial surgery so as its related complications can be prevented or treated.^5^ Although ROSO carries risk of retinal re-datachment and some other complications but all VR specialist agreed that once the objectives of temponade is achieved and retina become stable, silicone oil should be removed.

Studies report several techniques for ROSO including anterior approach through limbus and posterior through pars plana route. Conventional pars plana technique of ROSO include conjunctival peritomy followed by 20 G sclerotomy that need closure with sutures and 3-4 weeks for that discomfort to recover. Not only it take more time for ROSO with 20 G vitrectomy but also carries the risk of post operative wound leakage, retinal incarceration, higher intra ocular pressure (IOP)and choroidal detachment as well.^6^ With the advent of 23 G and 25 G transconjunctival sutureless vitrectomy systems (TSVS), vitreo retinal surgery entered into a new mico incisional era. These sutureless, self sealing, angled trans conjunctival sclerotomies had greatly minimized opening and closing times thus resulting quick recovery had reduced patients stress as compared to classic 20 G three port vitrectomy.^7^,^8^ Active ROSO through conventional 20 G instruments is easy while passive ROSO is quite difficult by 20 G system because sclerotomies made by micro vitreo retinal (MVR) knife tend to collapse. In 23 G system, rigid cannulae are passed into sclerotomy wound that keeps it open all the time so oil easily flow out passively. Moreover, passive ROSO is more controlled with less fluctuation of pressure and requires minimal maneuvering during procedure.^9^

The purpose of our study was to see the outcomes of passive ROSO through 23 gauge TSVS. To the best of our knowledge no such study has been conducted in our setup so we studied the efficacy and safety of 23 Gauge TSVS in terms of per operative and post operative difficulties and complications.

## METHODS

This prospective, descriptive interventional case series study was conducted from January 2011 to December 2014 at Ophthalmology Department Unit I, Dow University of Health Sciences and Civil Hospital Karachi. Seventy nine eyes underwent passive ROSO through 23 gauge TSVS under retrobulber anesthesia. All gender patients of all age group were enrolled after explaining pros and cons of the procedure and written informed consent was obtained. Only psuedophakic eyes having history of retinal reattachment surgery with silicone oil temponade were included in this study. Phakic and aphakic eyes were not enrolled. Eyes with corneal pathology or having any other retinopathy were excluded from this series. After complete history and ocular examination, indication for ROSO was established. Pre operative best controlled intra ocular pressure was recorded in each case.

Under all aseptic measures, the conjunctive was displaced 1-2mm cross wise in the direction of limbus at infero-temporal, supero-temporal and supero-nasal quadrants. Making an angle of 10 degree to sclera, three polyamide micro cannulae (trocar) of 23 gauge were inserted trans conjunctivally in the sclera at pars plana site. After advancing the trocar for 2mm, the angle was changed to 30 degree into the mid vitreous cavity. Through infero-temporal trocar site infusion line was connected with 70 to 90 cm bottle height. The silicone oil started to drain passively upon opening the infusion line through the two superior sclerotomy sites. The cannula was used to eliminate the last bubble of silicone oil. At the complete removal of silicone oil, retinal status was closely examined and fluid air exchange (FAX) was done. While removing micro cannulae, moderate pressure was applied at each sclerotomy site with a cotton applicator and eye patched for 24 hours after installing topical antibiotic (Moxifloxacin) drops. Endolaser photocoagulation was performed wherever required and silicone oil refilled where retinal detachment was found on follow up visits. Time taken for ROSO was measured by video recordings. Any per operative difficulty or complication were recorded.

The patients were examined on next day and follow up was scheduled on 1st week, one, three and six month interval post operatively. On each visit, complete ocular examination was performed, intraocular pressure (IOP) was noted and retinal status was checked.

Statistical analysis was done using SPSS version 17.0. The difference between pre and post operative IOP was compared by using two-tailed paired t-test and mean values with standard deviation were computed using difference of 95% confidence interval. Chi square test was applied for correlation of different variables. P-value of less than 0.05 was considered statistically significant.

## RESULTS

Out of 79 patients who underwent passive ROSO, 38 (48.1%) were males while 41 (51.9%) were females. Mean age of patients was 47.5±7.1 (sd) years while majority (53.2%) were in the age group of less than 45 years. The main indication for ROSO was glaucoma in 53 (67.1%) cases followed by emulsification and presence of oil in anterior chamber. The silicone oil viscosity of 1000 and 5000 centistokes (cs) was found in 70 (88.6%) and 9 (11.4%) cases respectively (Table-I).

**Table-I:** Descriptive data (n=79).

*Data*	*Frequency*
***Age***	
• Minimum	34 years
• Maximum	62 years
• Mean	47.56±7.18 (SD)
***Groups (Years)***	
• Less than 45	42 (53.2 %)
• 46 to 55	23 (29.1 %)
• 56 & above	14 (17.7%)
***Gender***	
• Male	38 (48.1%)
• Female	41 (51.9%)
***Indications for ROSO***	
• Glaucoma	53 (67.1 %)
• Oil Emulsification	16 (20.3 %)
• Oil in AC	10 (12.7 %)
***Silicone Oil Viscosity***	
• 1000 centistokes	70 (88.6 %)
• 5000 centistokes	09 (11.4 %)

The mean time interval between silicone oil temponade surgery and maneuver for ROSO was 5.05±3.09 (sd) months. Mean time taken for passive ROSO by 23 G TSVS was 7.31 ±2.41 (sd) minutes (Table-II).

**Table-II:** Silicone oil temponade duration and time taken for ROSO (n=79).

*Time interval between temponade surgery and ROSO*	*Frequency*
• Minimum	2 months
• Maximum	15 months
Groups (Months)	
• Less than 3	33 (41.8 %)
• 3 to 6	33 (41.8 %)
• More than 6	13 (16.5 %)
Mean interval = 5.05±3.09 SD months.	
***Time Required for ROSO***	
• Minimum	4 minutes
• Maximum	15 minutes
Groups (Minutes)	
• 4 to 7	62 (78.5 %)
• 8 to 11	10 (12.7 %)
• 12 to 15	07 (8.9 %)
Mean time required for ROSO is 7.31±2.41 SD minutes.	

SD = Standard Deviation.

The comparison of pre and post operative intra ocular pressure (IOP) shows statistically significant (p=0.000) decrease in IOP after ROSO. Mean pre operative IOP was 28.81±11.45 (sd) mmHg which declines to mean IOP of 19.54±4.38 (sd) mmHg after ROSO (Table-III).

**Table-III:** Pre Operative & Post Operative IOP Comparison (n=79).

*IOP*	*PRE OP*	*POST OP*	*P -Value*
< 20 mmHg	26 (32.9 %)	51 (64.6%)	< 0.000*
21 – 30 mmHg	16 (20.3 %)	24 (30.4 %)	
31-40 mmHg	19 (24.1 %)	04 (5.1 %)	
>41 mmHg	18 (22.8 %)	nil	
• Pre operative mean IOP = 28.81±11.45 SD			
• Post operative mean IOP = 19.54±4.38 SD			
• Reduction in mean IOP= 9.26±11.79 SD			

* P-value less than 0.05 is statistically significant,

IOP = Intra Ocular Pressure,PRE OP = Pre operative,POST OP = Post Operative

Hypotony (IOP < 5mmHg) was observed in 5 cases post operatively which normalizes within first two days of ROSO. During passive silicone oil removal, extrusion of trocar cannulae were observed in 7 (8.9%) cases which were reinserted back successfully. All the silicone oil was removed successfully and retina found flat during the surgery. In majority of cases retina remain flat after the surgery whereas retinal redetachment developed in 13 (16.5%) cases during first month of follow up period (Table-IV).

**Table-IV:** Per operative and post operative complications.

*Complications*	*Per operative*	*Post operative*
Extrusion of Trocar	7 (8.9%)	Nil
Retinal Re-detachment	N/A	13 (16.5%)
Hypotony	Nil	5 Recovered within two days
Glaucoma	N/A	28 (35.4%)
Wound leak	Nil	Nil

## DISCUSSION

Various techniques have been adopted for ROSO to combat the established complications of silicone filled eyes. Eckardt^10^ in 2005 introduced 23 gauge TSVS which offer angled self sealing tunnel sclerotomies maintained through trocar canulae throughout surgery period. As it uses firmer and sturdier instruments as compared with 25 G, it does not require a prolonged learning curve. Hence this system is now getting popularity among VR surgeons for ROSO as well.

Raised IOP is a frequent complication of silicone oil temponade. The incidence of glaucoma varies widely from 2.2% to 56% cases and is reversible in most patients after oil removal.^11^-^13^ In our series we observed 53 (67.1%) eyes with chronic glaucoma which stands main indication for ROSO. In eyes having silicone oil endotemponade and raised IOP, ROSO is a preferred maneuver compared to invasive filtration glaucoma surgery.^14^ In this study mean IOP reduction was 9.26±11.79 (sd) after ROSO which is statistically highly significant (P= 0.000). Similar results were noted in a study where 93.4% of patients had normalization of IOP after removal of silicone oil temponade.^15^

Patwardhan and colleagues^9^ reported 6.86 minutes mean time for ROSO with 23 G trocar canullae system. However Yildirim et al.^16^ noted approximated 9 minutes for passive ROSO while Kapran et al.^17^ mentioned 7.3 minutes for passive ROSO with 25 G micro canullae system. In our study by 23 gauge TSVS, majority of eyes with 1000 cs silicone oil temponade, we observed 7.3 minutes mean surgical time for passive ROSO which is comparable with other studies. We recorded relatively longer time i.e. 12 to 15 minutes in 7 (8.9%) cases of passive ROSO which had 5000 cs silicone oil temponade so we recommend passive ROSO in such cases as well.

Timing for ROSO has remained under debate since long time. Many VR surgeons now are of the opinion that once the objective of internal temponade is achieved, silicone oil must be removed to prevent or treat complication associated with it. Nagpal et al.^18^ observed that the duration of temponade did not have an effect on the incidence of retinal redetachment after silicone oil removal. In our study mean interval between silicone oil temponade and ROSO was 5.05±3.09 (sd) months. Decision of ROSO must be weighed against the possible risk of retinal detachment. Khurram et al.^19^ reported retinal redetachment in 38% eyes, whereas Jahangir K^20^ found same complication in 33.3% cases after first month of ROSO through pars plana route. Although mechanisms for redetachment after ROSO includes the opening of pre existing breaks, the posterior migration of an occult rhegmatogenous RD or formation of new breaks; but none of these can be blamed definitely.^2^ In our series, we encountered 13(16.5%) retinal redetachment after ROSO. All cases were found in first month of ROSO. Our results are comparable to Falkner et al. and Tan HS et al.^21^,^22^ who reported 17.4% and 19.2% cases of redetachment after ROSO respectively.

O’Reilly et al.^23^ reported transient hypotoy in 10 out of 39 cases while Lakhanpal et al.^7^ noted same in 2 out of 140 cases. We noticed post operative hypotony in 5 cases which normalized within two days. This transient hypotony can be due to choroidal detachment or wound leak. No wound leak or choroidal detachment was found post operatively in any of our 79 cases. We observed extrusion of micro cannulae in 7 (8.9%) eyes during surgery. However reinserted successfully but slippery trocar could be a problem in this technique. Further larger, controlled studies must be conducted to understand detailed outcomes of this technique.

Passive ROSO through 23 gauge TSVS is technically easy in scarred conjunctiva from prior retinal surgery and being suture less makes it comfortable to patients. Small scleral incisions (23g transconjunctival) are advantageous in preventing endophthalmitis because they are immediately covered by intact Tenon capsule with an overlying intact conjunctiva which prevent bacterial invasion in vitreous cavity. Aseptic measures like sterile preparation with povidine-iodine decrease the potential risk of vitreous infection during trocar insertion. Sutureless technique eliminates post operative inflammatory reaction resulting from irritation of exposed sutures material which offers more patient comfort and early rehabilitation.

## CONCLUSION

Passive removal of silicone oil with 23 G transconjunctival suture less vitrectomy system is safe and effective in terms of less per operative and post operative complications. In this simple technique, there is less tissue trauma, less inflammation and little time consumed so it provides more comfort to patients and surgeons as well.
